# Higher Volume at Time of Breast Conserving Surgery Reduces Re-Excision in DCIS

**DOI:** 10.1155/2011/785803

**Published:** 2011-03-09

**Authors:** J. H. Wolf, Y. Wen, D. Axelrod, D. Roses, A. Guth, R. Shapiro, J. Cohen, B. Singh

**Affiliations:** ^1^Department of Surgery, New York University Langone Medical Center, New York, NY 10016, USA; ^2^Department of Surgery, Johns Hopkins Medical Institutions, Baltimore, MD 21287, USA; ^3^Department of Pathology, New York University Langone Medical Center, New York, NY 10016, USA; ^4^Department of Biological Statistics and Computational Biology, Cornell University, Jthaca, NY 14853, USA

## Abstract

*Purpose*. The purpose of this study was to compare the surgical and pathological variables which impact rate of re-excision following breast conserving therapy (BCS) with or without concurrent additional margin excision (AM). *Methods*. The pathology database was queried for all patients with DCIS from January 2004 to September 2008. Pathologic assessment included volume of excision, subtype, size, distance from margin, grade, necrosis, multifocality, calcifications, and ER/PR status. *Results*. 405 cases were identified and 201 underwent BCS, 151-BCS-AM, and 53-mastectomy. Among the 201 BCS patients, 190 underwent re-excision for close or involved margins. 129 of these were treated with BCS and 61 with BCS-AM (*P* < .0001). The incidence of residual DCIS in the re-excision specimens was 32% (*n* = 65) for BCS and 22% (*n* = 33) for BCS-AM (*P* < .05). For both the BCS and the BCS-AM cohorts, volume of tissue excised is inversely correlated to the rate of re-excision (*P* = .0284). Multifocality (*P* = .0002) and ER status (*P* = .0382) were also significant predictors for rate of re-excision and variation in surgical technique was insignificant. *Conclusions.* The rate of positive margins, re-excision, and residual disease was significantly higher in patients with lower volume of excision. The performance of concurrent additional margin excision increases the efficacy of BCS for DCIS.

## 1. Introduction

Ductal carcinoma in situ (DCIS) is a noninvasive cancer that can be an obligate precursor of invasive ductal carcinoma (IDC). Since the early 1990s, reported survival outcome for DCIS is equivalent for breast conserving surgery (BCS) versus mastectomy [1, 2]. BCS is an oncoplastic procedure, in that its goal is to remove all DCIS, including a circumferential margin of noncarcinomatous tissue, commensurate with preserving as much of the normal breast tissue and appearance as possible.

While there is no consensus on a safe margin width in breast conservation therapy, margins may be categorized as positive if directly involved with cancer cells, as inadequate if less than 1-2 mm, and as negative if greater than 10 mm [3]. Multiple studies have shown that ipsilateral breast tumor recurrence (IBTR) is associated with positive margins in patients undergoing BCS [4–6]. Most recently, the Early Breast Cancer Trialists' Collaborative Group concluded that the avoidance of four local recurrences would avoid breast cancer-related death [7]. Thus, obtaining appropriate surgical margins is of paramount importance in determining long-term outcome.

Margin width is also one of four critical factors employed in the USC/Van Nuys prognostic index (UVPNI: age, lesion size, margin width, and pathologic classification) as a means of appropriately stratifying patients with DCIS into three separate treatment categories [8]. The UVNPI identifies those patients who may be treated without radiation and those who may require mastectomy. While based on a prospective non-randomization of patients, the UVPNI provides guidelines to improve patients' prognostic indices with additional surgery that increases margin width. Margin width is the only modifiable factor that may obviate the need for radiation and reduce the risk of recurrence. 

In an attempt to minimize the need for re-operation and maintain anatomic accuracy, many surgeons now obtain “additional margins” (BCS-AM) at the time of the initial surgery by shaving off a thin layer of tissue inside the surgical cavity in one or all of the six dimensions. This shaved tissue now serves as the “true” surgical margin. The efficacy of this approach in preventing re-excision and IBTR for IDC and DCIS has been addressed in several studies [9–14]. The added benefit of re-excising additional tissue at the time of initial surgery is the certainty of the location of the additional tissue in relation to the index cancer. The purpose of this paper is to compare BCS and BCS-AM procedures with respect to rates of re-excision for DCIS based on our institutional experience.

## 2. Materials and Methods

### 2.1. Case Accrual

Institutional Review Board approval was obtained to query the Department of Pathology database for all patients who underwent surgical management for DCIS from January 2004 to September 2008, with all cases reviewed by a single pathologist (B. Singh). The reports were reviewed for demographic, surgical, and histopathologic data, as well as reoperations.

**Figure 1:**
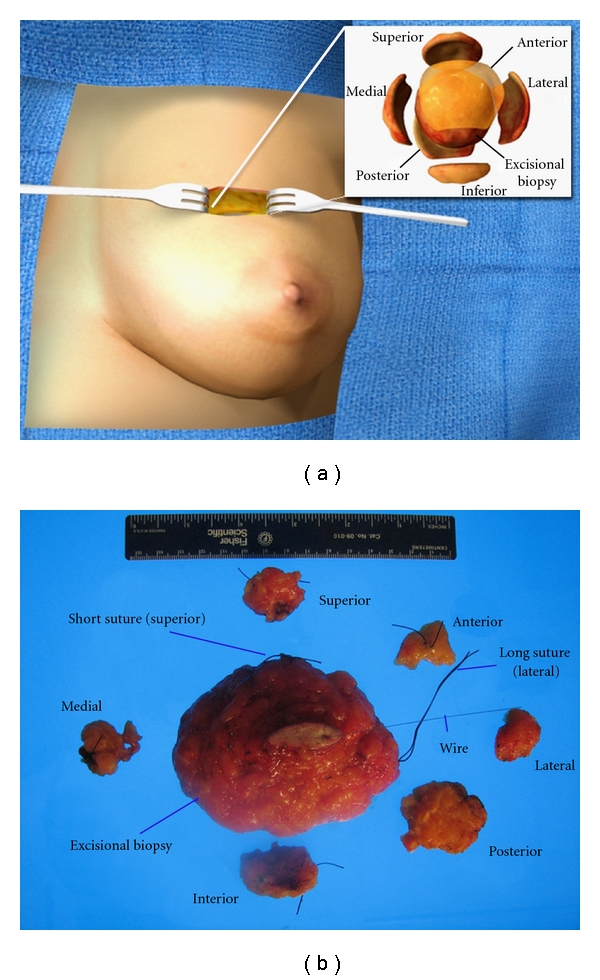
(a) Schematic for BCS-AM. The segmental excision surgical specimen is removed using wire localization. Additional margins can then be re-excised from each of the six faces of the cavity (anterior, posterior, superior, inferior, medial, and lateral). (b) BCS-AM surgical specimen. The total tissue obtained from the BCS-AM procedure includes the original localized lesion (shown with wire placement), as well as one or more additional margins from the six surfaces.

A group of 405 patients were retrospectively identified using patient records as having undergone surgical management for DCIS at our institution. In 201 patients (50%), standard BCS was performed (without removal of AM); in 151 patients (37%), BCS-AM was performed (Figure 1), the remaining 53 patients (13%) underwent total mastectomy (TM) as their initial surgical management.

Surgery was performed by thirteen surgeons in this study. However, the first four surgeons performed 86.6% of the surgical procedures in the study. Since the other nine surgeons did not perform a minimum of 30 surgeries, have a minimum cell size of five for BCS and BCS-AM surgeries, or perform a minimum of 10% of the surgeries in the total sample, we did not compare their surgical techniques. This maintains high statistical power levels of performed ANOVA and chi-squared tests. The four surgeons abstracted their patient charts for: (1) indications for surgery, including a palpable mass, calcifications, and mammographically or MRI-detected lesions, (2) surgical technique: localization wire or bracket and (3) indications for re-excision: mass, residual calcifications, or residual DCIS close to margins (Table 1(b)). An individual patient may have more than one indication for surgery. 

In general, sentinel axillary node dissection with or without formal axillary nodal dissection is not performed for cases of DCIS, as it is presumed to be localized disease.

### 2.2. Surgical Technique

#### 2.2.1. Surgeon A

For non-palpable lesions, a modified Kopans hook wire localizes the lesion in 2 views (including after a sonographic localization) and is performed in the Department of Breast Imaging by the radiologist. In the resection of superficial lesions, an ellipse of skin is included, and, for deep lesions close to the chest wall, fascia is included. Intraoperatively, at least a 1cm rim of normal tissue in all directions is taken. Electrocauterization and sharp dissection are both used to excise the lesion. The margins are marked accordingly with a long suture denoting the lateral margin and a short suture denoting the superior margin. A confirmatory digital specimen X-ray is taken in 2 views and the films are reviewed intraoperatively by the surgeon. The specimen is then sent to pathology as a fresh specimen. The breast imager subsequently reviews the specimen film.

At the discretion of the surgeon, additional margins are taken. They are oriented using a suture to mark the new true margin and secured to a telfa pad and sent to pathology as a fresh specimen.

#### 2.2.2. Surgeon B

For palpable lesions, excisions are performed with estimated margins of at least 1 cm and suture tagged for orientation. When any margin is measured in the pathology laboratory on immediate inspection as closer than the desired 1 cm, an additional segment is excised from the excision bed at that site. Superficial lesions are usually excised with an ellipse of overlying skin. Posterior lesions are excised down to and often including the underlying muscle fascia. Nonpalpable lesions, including microcalcifications and/or biopsy clips, are excised with hook wire localization and specimen radiographic control to insure complete excision. The hook wire localizer provides additional orientation, and any margins estimated as close on specimen radiography, or by direct inspection for mass lesions or clips in the pathology laboratory, are treated by additional excision of the appropriate cavity margin sites.

#### 2.2.3. Surgeon C

For a palpable mass, the lesion is excised with an approximate 1 cm margin of normal tissue, then the specimen is examined for adequacy of margins and oriented with sutures. Additional margins are routinely excised, the extent of which is influenced by the original specimen. Superior, medial, inferior, lateral, anterior, and posterior margins are excised, oriented with sutures to indicate new margin, and sent individually fresh to pathology.

For radiologic-detected disease, following needle localization of a mass lesion, calcifications or a clip, an excision is performed around the lesion and localizing guidewire. If the lesion is superficial, an ellipse of overlying skin is removed; the pectoralis fascia is usually taken as the deep margin. Then, additional margins are routinely excised and sent to pathology, with the extent of excision based on inspection of the specimen and the specimen radiograph. If the radiologic abnormality is not included in the needle-localization specimen, or only partially included, the surgical site is first re-excised and the new specimen X-rayed. If this includes the lesion, routine margins are then sent as above.

#### 2.2.4. Surgeon D

Using a 10-blade scalpel, the segment of breast tissue containing the lesion (palpable or area localized by a hook wire) is excised with 1 cm margins of grossly uninvolved tissue and oriented with sutures. A Babcock clamp and 10-blade scalpel are then used to obtain additional margins of at least 1 cm in the superior lateral, inferior medial, anterior, and posterior planes. For lesions subadjacent to the skin, a small ellipse of overlying skin is included as an anterior margin. Deep lesions lying on the pectoralis major fascia are excised en bloc with the underlying fascia. A thin superficial layer of underlying muscle is obtained to augment the deep posterior surgical margin. All true margins are marked with sutures.

### 2.3. Pathological Examination

The excision specimens were measured in three dimensions and oriented, and six aspects were inked with different colored inks that can withstand histological processing. The specimen was serially sectioned along the long axis, and biopsy site and clip, if present, were identified. All cases were submitted entirely for microscopic examination. Additional margin specimens were measured in three dimensions and oriented with one suture identifying the true margin, which was typically inked black, with the opposite margin inked blue. The additional margins were also submitted entirely for microscopic examination. 

Volume of each specimen was calculated by multiplying the three dimensions of each specimen, noted on gross examination. The extent of DCIS was reported as the number of slides with DCIS and total number of slides for the case.

If DCIS was present >10 mm from a margin, it was considered negative and this distance was not reported in the final pathology report. If DCIS was <10 mm from a margin, the closest distance to the margin was reported. Microscopic examination of DCIS included assessment of morphologic subtype, nuclear grade, necrosis, multifocality, and calcifications. Multifocality is defined as presence of DCIS in a section prepared from more than one block of the specimen, which is the standard definition used by NSABP [1]. The volume of DCIS was assessed on microscopic examination as number of slides with DCIS over the total number of slides. Immunohistochemical stains for estrogen receptor (ER) (SP1 antibody, Ventana) and progesterone receptor status (PR) (1E2, Ventana) were performed for all cases and reported as a percentage. Microscopic examination of re-excisions used the same parameters as for primary excision. The final pathology report included a summary of pathological variables, including the margin status and biomarkers (ER and PR).

## 3. Results

### 3.1. Re-Excision Rate for BCS-AM Is Significantly Lower than for BCS

Patients who underwent BCS (*n* = 201), BCS-AM (*n* = 151), or TM (*n* = 53) for DCIS between 2004 and 2008 at NYUMC were compared by clinicopathological variables and were found to be similar in age, grade and size of DCIS, necrosis, multifocality, calcifications, and ER/PR status (Table 1(a)). Patients who underwent BCS-AM instead of BCS had a 10% lower incidence of positive or <1 mm margins (*P* < .05) and a 24% higher incidence of widely negative margins (>10 mm; *P* < .05). Accordingly, rates for re-excision were significantly (*P* < .0001) lower in the BCS-AM cohort compared to the BCS alone (BCS-AM: 40%, BCS: 64%). Residual DCIS in re-excision specimens was also significantly lower (*P* < .05) when AM was obtained (BCS-AM: 22%, BCS: 32%). Residual DCIS was found in roughly half of the re-excisions for the BCS group (50%) compared to the BCS-AM group (54%), which is statistically significant (*P* < 005).

### 3.2. Pathological Predictors for Re-Excision

Seven variables (necrosis, multifocality, calcification, ER, PR, total volume, BCS, or BCS-AM) and their cross-products with BCS/BCS-AM (six more variables) were analyzed to see which could be used to predict the response variable, the probability that re-excision surgery will need to be performed (Table 2(a)). The optimal model (*P* ≤ .0001) for this response variable was found by comparing a series of reduced multivariate logistical regressions using *G* ^2^ test statistics and log likelihoods. After completing this analysis, the optimal model to predict the probability of the occurrence of a re-excision surgery contained four significant predictors: BCS/BCS-AM (*P* ≤ .0001), multifocality (*P* = .0002), total volume (*P* = .0284), and ER (*P* = .0382) (these *P* values come from the parameter estimates in the optimal reduced model) (Table 2(b)). The variables necrosis (*P* = .5300), calcification (*P* = .8007), and PR (*P* = .9952) were found to be insignificant predictors and were, therefore, not in the optimal model (these *P* values come from the parameter estimates in the original full model).

### 3.3. Higher Excision Volume Is Related to Lower Rate of Re-Excision

For both the BCS and the BCS-AM cohorts, volume of tissue excised is inversely correlated to the rate of re-excision (Table 3). In the BCS group, patients with and without re-excision had total volumes of 77.8 cm^3^ and 100.3 cm^3^, respectively, (*P* = .067). Patients who underwent BCS-AM with and without re-excision had total volumes of 98.7 cm^3^ and 127.2 cm^3^, respectively, (*P* = .281). The volume of additional margins with and without re-excision was 28.9 cm^3^ and 26.3 cm^3^, respectively (*P* = .44). The volume of excision for all patients who did not undergo re-excision was 114.96 cm^3^ and those who did was 79.6 cm^3^. Using a two sample *t*-test, a significant difference in total volume was found when comparing BCS and BCS-AM surgery (*P* = .0003). Furthermore, when comparing surgery type with respect to re-excision rates using a series of two-sample *t*-tests, a significant difference in volume size was found between BCS surgery that required re-excision surgery and BCS-AM that did not require re-excision surgery (*P* ≤ .0001). A subset analysis of patients with positive or close margins (<1 mm) only found the volume of excision specimen not statistically significant (*P* = .713).

The patients were stratified by margin status, regardless of the procedure performed, to examine the relationship between negative margins and total volume (Table 4). The five margin size categories' total volumes were compared using a series of two-sample *t*-tests. A significant difference in total volume was observed when comparing positive margins with all the other margin sizes. In this analysis, widely negative margins (>10 mm) correlated with the highest average total excision volume (130.4 cm^3^) and positive margin status correlated with the lowest (61.2 cm^3^) average total excision volume. Width of margin, represented by five different groupings (positive, 1-2 mm, 3–5 mm, 6–9 mm, negative), and total excisional volume were linearly related (*r* ^2^ = 0.931).

### 3.4. Surgeon (Surgical Technique) As a Variable for Rate of Re-Excision

Individual surgeons were also compared to determine if variation in surgical techniques (BCS and BCS-AM) was a significant variable for rate of re-excision. Four surgeons performed 86.6% of the surgical procedures. Nine surgeons did not perform a minimum of 30 surgeries, have a minimum cell size of 5 for BCS and BCS-AM surgeries, or perform a minimum of 10% of the surgeries in the total sample. To keep the statistical power of the ANOVA and the chi-squared test high (1-*β* > 0.8), only the first four surgeons were compared. 

The surgeons' techniques were compared by individual re-excision surgery rates relative to three major variables: type of surgery (BCS/BCS-AM), total volume, and margin size. The rate of re-excision surgery after BCS (*P* = .141355) and BCS-AM (*P* = .186487) was compared using the chi-squared test and no significant difference was demonstrated among the surgeons (Table 5(a)). For specimens that required a re-excision, the specimen volumes for each surgeon were compared using a one-way Analysis of Variance test, and no significant difference was found between the surgeons (*P* value of *F* statistics =.156) (Table 5(b)). Individual surgeon's rate of re-excision surgery was compared by margin size (Table 5(c)). Each surgeon was compared to the three other surgeons using chi-squared tests or *F* statistics from an ANOVA, and no significant difference was found.

## 4. Discussion

In this study, we evaluated the efficacy of simultaneous additional margin excision at the time of initial BCS in reducing the rate of surgical re-excision. Our data show that BCS-AM, compared to BCS, had a 10% lower incidence of positive or <1 mm margins (*P* < .05) and a 24% higher incidence of widely negative margins (>10 mm; *P* < .05). 

Accordingly, rates for re-excision were significantly (*P* < .0001) lower in the BCS-AM cohort than the BCS alone (BCS-AM: 40%, BCS: 64%). Statistical analysis revealed multifocality (*P* = .0002), total volume (*P* = .0284), and ER (*P* = .0382) to be significant predictors of re-excision in our population. In this study, total excision volume correlated with margin width regardless of procedure and variation in surgical technique and was inversely correlated with re-excision rate for both BCS and BCS-AM. In cases of re-excision, the total volume of tissue excised with BCS-AM is higher than that with BCS, and the dataset is limited in this regard. Variation in surgical technique was not found to be a significant variable. As such, our analyses emphasize the importance of total excision volume in successful BCS procedures and validate BCS-AM as an effective means of retaining anatomical accuracy and reducing the incidence of a second re-excision procedure. The major concern in the management of patients in our setting was the need for subsequent excision where margin widths were neither widely negative (>10 mm) nor definitively positive (<1 mm). The decision to re-excise in these cases was the presence of DCIS in the vicinity of the margin for all four surgeons and was performed without exception for margins <1 mm. Despite a generally uniform approach to the BCS and BCS-AM technique at our institution, we recognize that surgeon variability is a potential confounder in our study. To address this, we compared the four major surgeons involved in our case cohort (representing 87% of cases) and found they were not statistically different with respect to our outcome measures.

Taking additional margins provides a technical benefit to the pathologist. Additional margins provide more accurate spatial orientation of the surgical specimen compared to inking by a pathologist, as the in situ anatomy is at variance from the excision specimen. There are also instances in which inked specimen margins provide false negative data that an additional margin would in effect correct. A recent study by Povoski et al. found that half of their additional margin specimens that were positive for carcinoma (13/24) had a negative excision specimen [13]. We also, however, noted the reverse effect in our study, as did Cao et al. [11], in which a re-excision performed for a positive margin was negative in a number of cases. Some possible explanations for this include small residual tumor volume, tissue necrosis at the new margin, or tissue friability as a result of processing. Interestingly, Rizzo et al. did not find additional margin sampling to be significantly burdensome or costly to pathologists [14]. Additional margins should represent entire aspects of the excision cavity; however, currently, there is no reproducible and effective method to ascertain spatial accuracy of additional margins. 

This analysis has some limitations inherent in a retrospective cohort. The inclusion criteria (indication for surgery, e.g., palpable mass, non-palpable mass, calcifications, mammographic, sonographic, or MRI lesion) were not controlled for. The endpoint of this study is the rate of re-excision. In addition, our study does not provide long-term followup and outcome; thus, we cannot comment on how BCS and BCS-AM compare with respect to disease-free survival. 

Shaving additional margins for excision of invasive and noninvasive carcinoma has already been shown to be effective in reducing re-excision rates and reducing margin positivity for early stage breast cancer [9, 11–15] Cao and colleagues [11] retrospectively examined 126 patients with either DCIS (*n* = 23) or IDC (*n* = 103), all of whom underwent BCS-AM surgery that was similar in methodology to our series (Figure 1). Among their patients, 103 had excisional specimens with histologically positive margins; yet, of these patients, only 52 (50.5%) had detectable carcinoma present on the additional margins. The investigators identified two pathological features, namely, “large tumor size” and “presence of a DCIS component” as significantly correlated with histologically positive lumpectomy margins (in which carcinoma is <2 mm from surface). By examining the pathology from both the original lumpectomy margins and the additional margins, they determined that 59% of margins were histologically negative as a direct result of removing additional margins. 

A study by Janes and colleagues [9] found similar efficacy in the surgical approach, linking systemic superior and inferior cavity shavings (SSICS) to reduction in close margins and re-excision rates. SSICS is a technique identical in principle to the “additional margins” approach employed in our study. The investigators prospectively compared two groups of patients. The first (*n* = 106) was a group with IDC or DCIS who underwent BCS, followed by pathological examination of the specimen. If margins were deemed <10 mm, then additional margins were removed as needed. In the second group, patients underwent SSICS initially. The group receiving SSICS had a reduced rate of close margin (18/106 versus 8/111 or 17% versus 7%) and a reduced rate of re-excision.

Several other retrospective analyses are notable as well. In a separate retrospective study, Huston et al. [15] divided a group of 171 patients with IDC/DCIS into three subdivisions, based on number of additional margins obtained (4–6, 1–3, and 0), and compared their respective rates of re-excision. Re-excision was performed if any margin was found to contain carcinoma within 2 mm of its border edge. They found that the group with 4–6 margins had a reoperative rate of 17.7% compared with 32.5% and 38.7% for the other two, respectively. Interestingly, the three groups also had different volumes, correlating with the reoperative rates, as was the case in our data (129.19 cm^3^, 46.04 cm^3^, and 37.44 cm^3^, resp.) In another analysis of 125 DCIS and IDC lumpectomy specimens, Jacobson et al. report that by taking additional margins re-excision was avoided in 49% of their patients for whom it otherwise would have been necessary [12]. Rizzo and colleagues reported 320 patients who underwent BCS (*n* = 199) or BCS-AM (*n* = 121) for either DCIS or IDC and observed an increase in negative margins (85.1% from 57.2%) and a decrease in re-excisions with additional margins. This study did not find the effect to be significant for DCIS when it was analyzed separately [14]. 

As discussed above, prior reports suggest that excising additional margins reduces both positive margin status and rate of re-excision for IDC and IDC with a DCIS component. Our data suggest that this effect is also true for cases of pure DCIS and that the approach is successful because of higher overall excision volumes. The importance of this finding is highlighted by the published literature linking volume of excision to rates of recurrence. A report by Vicini et al. reviewed 146 patients who received BCS for DCIS and used a multivariate analysis to identify factors associated with reduced local recurrence. They concluded that margin width and volume were both independent predictors of outcome [16]. Similarly, Hwang et al. found that elderly patients with DCIS seen at their institution were more likely to have large volume excisions as compared to younger patients. They propose this as one possible reason for lower recurrence rates in the elderly group [17]. Surgeons may be more reluctant to excise larger volumes of tissue in younger patients with DCIS because of potential cosmetic deformity.

## 5. Conclusion

Our studies found multifocality to be strongly associated with re-excision in agreement with the National Surgical Adjuvant Breast and Bowel Project (NSABP B-17), in which multifocality was predictive of positive margins [1]. DCIS is generally not a palpable lesion, making multiple foci more challenging to identify during surgery, even with wire-guided radiological assistance. These multiple foci may represent an artifact of pathological processing, where a three-dimensional disease process is visualized in a two-dimensional format. Multiple foci of DCIS in the same quadrant probably represent the same genetic profile, as has been demonstrated for invasive carcinoma [18]. Our data suggest that excising additional margins for DCIS increases the total average excision volume and decreases the incidence of re-excision.

## Figures and Tables

**Table:** (a) Comparison of patients who underwent BCS, BCS-AM, and TM procedures.

	BCS	BCS-AM	TM
No. of patients	201	151	53
Age (mean)	58	59	54
Age (median)	58	58	54
DCIS grade			
Grade I	17	10	5
Grade II	88	53	15
Grade III	96	88	33
DCIS size			
<1 cm	67	44	9
1-2 cm	37	22	6
2–5 cm	27	19	9
>5 cm	9	1	2
N/A	61	65	27
Necrosis			
Yes	124	106	40
No	77	45	13
Calcifications			
Yes	147	109	37
No	53	42	16
Multifocality			
Yes	112	101	34
No	89	50	19
ER			
71–100%	112	83	19
41–70%	8	10	3
11–40%	5	2	0
0–10%	29	26	23
N/A	47	30	8
PR			
71–100%	22	15	8
41–70%	15	13	5
11–40%	24	16	4
0–10%	93	76	28
N/A	47	31	8
ER/PR			
Both 71–100	29	19	8
Both <10	19	25	19
Margin**			
Positive, <1 mm	117	61*	1
1-2 mm	23	9*	0
3–5 mm	17	17	0
6–9 mm	6	4	0
≥10 mm	37	60*	52
Re-excision	129	61***	2
Residual DCIS	65	33*	0

BCS: breast conserving surgery; BCS-AM: BCS plus additional margin; TM: total mastectomy. *P < .05, **P total <.0001, ***P < .0001.

**Table:** (b) Indications for surgery and re-excision, categorized by surgeon.

	Surgeon A	Surgeon B	Surgeon C	Surgeon D	Total	%
Indication for surgery						
Palpable mass	5	10	4	5	24	6.8
Nonpalpable mass	14	0	0	0	14	4.0
Calcifications	55	96	59	67	277	78.7
Mammographic lesion	53	15	59	70	197	56.0
Sonographic lesion	8	0	6	0	14	4.0
MRI lesion	6	11	7	1	25	7.1

Surgical procedure						
Needle localization	60	85	40	67	252	71.6
Surgery bracket	10	2	4	0	16	4.5

Indication for re-excision						
DCIS at margin	6	18	11	13	48	31.4
Clearance <1 mm	22	28	13	16	79	51.6
Clearance <2 mm	9	4	4	2	19	12.4
Clearance <3 mm	5	1	1	0	7	4.6

**Table:** (a) Comparison of patients who did not require re-excision to those who underwent re-excision.

	BCS		BCS-AM		TM	
Re-excision	No	Yes	No	Yes	No	Yes
No. of patients	72	129	90	61	51	2

Re-excision	No	Yes	No	Yes	No	Yes
No. of patients	72	129	90	61	51	2

Necrosis						
Yes	39	82	62	44	37	2
No	33	45	28	17	13	0
N/A	0	2	0	0	0	0
Calcifications						
Yes	51	95	67	41	36	2
No	21	30	23	19	15	0
N/A	0	4	0	0	0	0
Multifocality						
Yes	36	74	53	48	34	1
No	36	50	37	13	17	1
N/A	0	4	0	0	0	0
ER						
71–100%	40	72	50	34	21	0
41–70%	2	6	7	3	2	0
11–40%	3	2	1	1	3	0
0–10%	7	20	11	14	16	2
N/A	20	22	21	9	9	0
PR						
71–100%	8	13	10	5	7	0
41–70%	7	8	6	7	5	0
11–40%	2	22	8	8	5	0
0–10%	35	57	44	32	25	2
N/A	20	22	22	9	9	0
ER/PR						
Both 71–100%	8	16	10	8	8	0
Both 0–10%	7	16	11	14	15	2

**Table:** (b) Statistical significance of predictors for re-excision.

Parameter	Prob > Chi Sq
BCS	*<.0001
Multifocal	*.0002
Total volume	*.0284
ER	*.0382
Necrosis	.5300
PR	.9952
Calcifications	.8007

*Significant predictor of re-excision. The *P* values of the chi-squared test show the significance of each parameter as a predictor of the response variable, rate of re-excision surgery. If the *P* value is less than  .05, then the parameter is considered significant and is a good predictor in the model for re-excision.

**Table:** (a) Comparison of the average total volume of BCS and BCS-AM surgery relative to re-excision surgery.

	Re-excision			
	No	Yes	*P*	Total
BCS	100.3 (*n =* 79)	77.8 (*n =* 122)	*.067	86.6 (*n =* 201)
BCS-AM	127.2 (*n =* 95)	98.7 (*n =* 56)	*.281	116.6 (*n =* 151)

Total	114.9 (*n =* 174)	79.6 (*n =* 178)	*.003	

				**<0.001

AM alone	26.3 (*n =* 95)	28.9 (*n =* 56)	*.444	24.6 (*n =* 151)

BCS-AM: all resected tissue, including additional margins. AM alone: volumes of additional margins taken with BCS for BCS-AM samples. The volumes of each category were compared relative to re-excision surgery using the *P* values from two-sample *t*-tests with unequal variances. *P value reflects difference between “yes” and “no” for each listed group. **P value reflects difference between “total BCS” and “total BCS-AM”.

**Table:** (b) Comparison of average total volume (cm^3^) for BCS and BCS-AM surgery relative to re-excision surgery using data only from the positive and close margin (<1 mm ) cohort.

	Re-excision			
	No	Yes	*P*	Total

BCS	81.5 (*n* = 19)	72.9 (*n* = 98)	.773	74.3 (*n* = 117)
BCS-AM	97.6 (*n* = 17)	100.961 (*n* = 44)	.907	100.0 (*n* = 61)

Total	89.0 (*n* = 36)	81.6 (*n* = 142)	.713*	83.1 (*n* = 178)
**< 0.079				

*P value reflects difference between “yes” and “no” for each listed group. **P value reflects difference between “total BCS” and “total BCS-AM”.

**Table 4:** Volume of excision relative to the margin status.

Margins (*n =* 351)	Total avg volume (cm^3^)
Positive (*n* = 178)	83.1
1-2 mm (*n* = 32)	79.4
3–5 mm (*n* = 34)	113.5
6–9 mm (*n* = 10)	112.9
Negative (*n* = 97)	130.4
*F* statistic = 3.48	*P* value = .008*

This table compares the total average volume of each of the five categories using *P* values of two-sample *t *tests with unequal variances, after the data experienced a natural log transformation. *P value represents *P* value of *F* statistic in ANOVA test.

**Table:** (a) The rate of re-excision for each surgeon compared to type of surgery (BCS/BCS-AM).

Surgeon*	BCS			BCS-AM		
	# Re-exc	Total	Percent	# Re-exc	Total	Percent
A	24	35	68.57%	17	31	54.84%
B	18	37	48.65%	11	39	28.21%
C	41	78	52.56%	9	20	45.00%
D	20	22	90.91%	12	43	27.91%
Summary	chi squared		*P* value	chi squared		*P* Value

Total	5.455184		0.141355**	4.807951		0.186487**

*Only top four surgeons were chose to keep power of the test above 0.80.

**Table:** (b) The rate of re-excision for each surgeon compared by average total volume of excision (cm^3^).

Surgeon*	A	B	C	D
Volume	80.94	97.06	102.88	57.40
St Dev	72.45	86.98	124.09	51.14
*N*	41	29	50	32
*F* statistic.		1.77	*P* value	0.156

*Only top four surgeons were chose to keep power of the test above 0.80.

**Table:** (c) The rate of re-excision for each surgeon compared relative to margin size.

Margin size	Surgeon A		Surgeon B		Surgeon C		Surgeon D		Total		
	Re-exc	*n*	Re-exc	*n*	Re-exc	*n*	Re-exc	*n*	Re-exc	*n*	*P* value

Pos	25	32	25	35	42	48	29	33	121	148	.836699
1-2 mm	9	10	3	7	2	5	2	5	16	27	.468393
3–5 mm	6	9	0	7	2	8	1	2	9	26	.141999
6–9 mm	0	5	1	4	0	1	0	0	1	10	.472367
Neg	1	10	0	23	4	34	0	24	5	91	.145699

Total	40	56	29	53	46	62	32	40	147	211	
